# Modifying SnS_2_ With Carbon Quantum Dots to Improve Photocatalytic Performance for Cr(VI) Reduction

**DOI:** 10.3389/fchem.2022.911291

**Published:** 2022-06-22

**Authors:** Weidong Li, Jianping Qiu, Haihong Jin, Yuanyuan Wang, Dandan Ma, Xinxiang Zhang, Huayun Yang, Fangyuan Wang

**Affiliations:** ^1^ Zhejiang Normal University Xingzhi College, Jinhua, China; ^2^ Hangzhou Normal University Qianjiang College, Hangzhou, China; ^3^ Zhejiang Hongyi Environmental Protection Technology Co., Ltd., Hangzhou, China; ^4^ Environmental Engineering Corporation of Zhejiang Province, Hangzhou, China; ^5^ Zhejiang Tianchuan Environmental Science and Technology Co., Ltd., Hangzhou, China; ^6^ Zhejiang Normal University, Jinhua, China

**Keywords:** photocatalyst, SnS_2_, Cr(VI), carbon quantum dots, photoreduction

## Abstract

The photoreduction for hazardous Cr(VI) in industrial wastewater has been considered a “green” approach with low-cost and easy-to-go operation. SnS_2_ is a promising narrow bandgap photocatalyst, but its low charge carrier separation efficiency should be solved first. In this work, N-doped carbon quantum dots (CQDs) were prepared and loaded onto SnS_2_ nanoparticles *via* an *in situ* method. The resulting composite samples (NC@SnS_2_) were characterized, and their photocatalytic performance was discussed. SnS_2_ nanoparticles were obtained as hexagonal ones with a bandgap of 2.19 eV. The optimal doping level for NC@SnS_2_ was citric acid: urea:SnS_2_ = 1.2 mmol:1.8 mmol:3.0 mmol. It showed an average diameter of 40 nm and improved photocatalytic performance, compared to pure SnS_2_, following a pseudo-first-order reaction with a kinetic rate constant of 0.1144 min^−1^. Over 97% of Cr(VI) was photo-reduced after 30 min. It was confirmed that modification of SnS_2_ with CQDs can not only improve the light-harvesting ability but also stimulate the charge separation, which therefore can enhance the photoreactivity of SnS_2_ toward Cr(VI) reduction. The excellent stability of NC@SnS_2_ indicates that it is promising to be practically used in industrial wastewater purification.

## Introduction

Due to the increasing industrial activities, more and more wastewater needs to be treated since it contains heavy metal ions, toxic matters, and hazardous compounds (Lofrano et al., 2013). Among the highly concerned metal ions in industrial wastewater, Cr(VI) attracts public attention since it is easily assimilated by the human body with accumulation effect, exerting harmful effects to the liver. Liver cancer has been related to the excess uptake of Cr(VI). As a consequence, Cr(VI) total amount is listed as one of the quality control quotas for industrial wastewater (Al Nafiey et al., 2017). Among the multiple treatment approaches for industrial wastewater, such as water splitting, CO_2_ reduction, N_2_ fixation, and full adsorption/removal, photoreduction seems like an economic approach with easy-to-go operation and acceptable performance (Santosa et al., 2008; Ji et al., 2015; Shi et al., 2018; Tasbihi et al., 2018; Wang et al., 2018). For example, Sun and coworkers reported the adsorption and removal performance of the yttrium-based metal-organic framework, which allows efficient removal of Sb(VI) in water (Li Q. et al., 2022). Liu and Yang reported a carnation flower-like Bi_2_O_2_CO_3_ photocatalyst for the photoreduction of Cr(VI) (Li L. et al., 2022). Here, with the help of photocatalysts, Cr(VI) is reduced to Cr(III) which is considered less harmful to the human body.

For most reported photocatalysts, high excitation energy in the UV region is required since they are wide bandgap semiconductors. As a consequence, the application of these wide bandgap photocatalysts needs to be assisted by UV-radiation when processing wastewater. To explore narrow bandgap photocatalysts which are expected to be excited by natural solar spectrum, metal sulfide semiconductors have been intensively explored (Azimi and Nezamzadeh-Ejhieh, 2015; Xiao and Jiang, 2017; Yan et al., 2017; Li et al., 2018). For example, Lv and coworkers have reported a series of photocatalysts based on Bi_2_O_2_CO_3_ and Bi_2_WO_6_@Bi_2_S_3_ with visible photoreactivity for NO oxidation and brilliant red X-3B dye (X3B) (Huang et al., 2018; Yang et al., 2019). These works spark the exploration for narrow bandgap photocatalysts. Among these metal sulfide candidates, tin disulfide (SnS_2_) seems attractive due to its perfect spectral matching with solar spectrum, low cost, and more importantly, low bio-toxicity (Cheng et al., 2017). A problem faced by SnS_2_-based semiconductors is their low photocatalytic efficiency. To solve this problem, co-catalysts are doped into SnS_2_, aiming at a higher separation/transportation efficiency of photogenerated charge carriers, so that the photocatalytic efficiency of SnS_2_ can be improved (Guo et al., 2016; Jing et al., 2018).

Among the various proposed photosensitizers for photocatalysis, carbon quantum dots (CQDs) have drawn much research attention owing to their virtues of chemical stability, bio-compatibility, economic synthesis, and excellent photoactivity, along with their promising photoelectronic and photocatalytic performance (Zhang et al., 2012; Jiang et al., 2015; Yu et al., 2016). For CQDs smaller than 10 nm, they are composed of sp^2^/sp^3^-hybridized C atoms, with a narrow bandgap covering the whole visible region and even the near-infrared region. In addition, their optoelectronic property, including their bandgap, can be conveniently modified by chemical modifications, offering various kinds of CQDs to meet the requirement for photocatalytic application (Yu et al., 2016).

**SCHEME 1:**
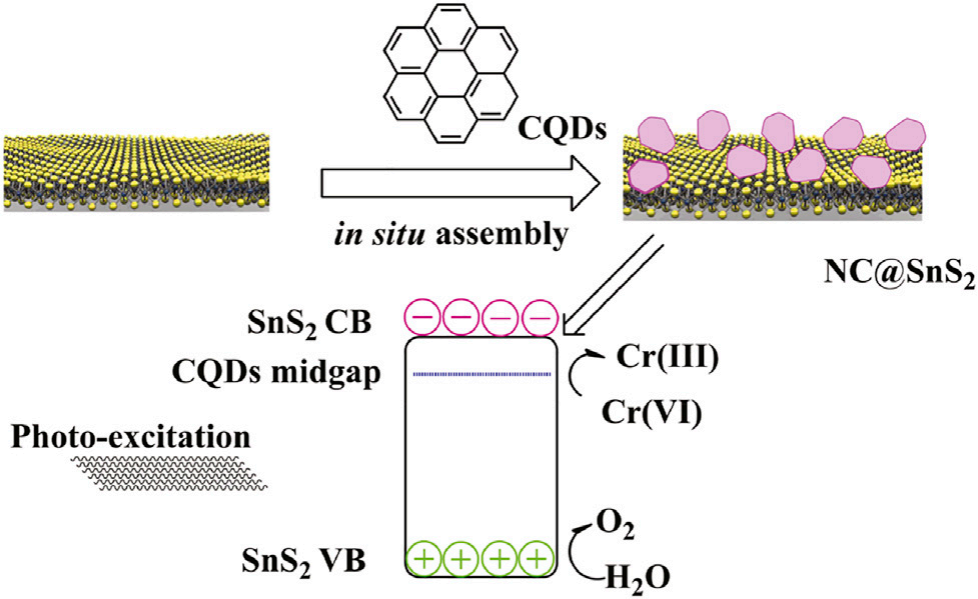
Synthetic and working strategy of NC@SnS_2_.

Guided by the aforementioned consideration, in this work, we intend to prepare N-doped CQDs and modify SnS_2_ with these N-doped CQDs. Here, the doped N atoms in CQDs are supposed to offer a midgap for the photogenerated charge carriers in SnS_2_ semiconductors, so that the electron mobility can be improved, which promotes the separation and transportation of photogenerated charge carriers. As a consequence, improved photocatalytic efficiency from the resulting composite structure (denoted as NC@SnS_2_) is expected. A schematic presentation for the synthetic strategy of NC@SnS_2_ is shown in Scheme 1. A full characterization on NC@SnS_2_ is performed, and corresponding photocatalytic performance for Cr(VI) photoreduction is discussed as well.

## Experimental Details

### Equipment and Reagents

All chemical reagents used in this work were AR-grade ones, including SnCl_4_, thioacetamide (TAA), citric acid (CA), urea (UA), and Nafion. Micromorphology analysis was performed using a Hitachi S4800 (SEM) microscope (Hitachi, Japan), an Oxford X-Max80 field emission scanning electron microscope (JSM-7800, JEOL), a Tecnai F20 (HRTEM), and a Talos F200X (FEI). IR, Raman, and XPS spectra were recorded on a Nicolet iS50 spectrophotometer (KBr method), a Renishaw inVia Raman microscope (excitation = 532 nm), and an ESCALAB250 X-ray Photoelectron spectrometer, respectively. Zeta potentials were determined by electrophoretic light scattering with a zeta sizer (nano series, Malvern, UK). XRD diffraction data were collected on a Rigaku MiniFlex Ultima IV diffractometer (Cu Kα source, *λ* = 1.5418 Å) with an operating voltage of 40 kV and a current of 15 mA. UV-Vis diffuse reflectance spectra (DRS) were recorded using a Hitachi U-4100 spectrophotometer equipped with an integrating sphere. UV-Vis absorption spectra were obtained using a Hitachi UV-2450 UV-Vis spectrophotometer. Electrochemical parameters were determined by a lock-in-based surface photovoltage (SPV) and an electrochemical workstation (CHI 760E).

### Synthesis of SnS_2_ Nanoparticles

SnS_2_ nanoparticles were synthesized as follows: a homogeneous solution was prepared by mixing SnCl_4_ and TAA with deionized water (molar ratio = 1:3 in 70 ml). This solution was sealed in a Teflon autoclave and heated at 190^o^C for 6 h. The solid product was separated by centrifugation (×4,000 rpm) and washed by H_2_O/EtOH (1:1). Before usage, the as-synthesized SnS_2_ nanoparticles were activated at 60^o^C for 12 h.

### Synthesis of NC@SnS_2_ samples

NC@SnS_2_ was synthesized by an *in situ* assembly method described as follows. A homogeneous solution was prepared by mixing CA and UA with deionized water (in 25 ml). The previously obtained SnS_2_ nanoparticles (3 mmol) were added and stirred for 30 min (×600 rpm). This suspension was sealed in a Teflon autoclave and heated at 160^o^C for 4 h. After natural cooling, EtOH (50 ml) was added. The solid product was separated by centrifugation (×4,000 rpm) and washed by H_2_O/EtOH (1:1). The obtained solid powder was dried at 80^o^C for 12 h and denoted as NC2-1@SnS_2_ (CA:UA = 0.30 mmol:0.45 mmol), NC2-2@SnS_2_ (CA:UA = 0.50 mmol:0.75 mmol), NC2-3@SnS_2_ (CA:UA = 0.90 mmol:1.35 mmol), NC2-4@SnS_2_ (CA:UA = 1.2 mmol:1.8 mmol), and NC2-5@SnS_2_ (CA:UA = 1.50 mmol:2.25 mmol).

By varying CA:UA ratio, two more NC@SnS_2_ samples were synthesized for performance comparison and denoted as NC1@SnS_2_ (CA:UA = 1.2 mmol:3.6 mmol) and NC3@SnS_2_ (CA:UA = 1.2 mmol:1.2 mmol).

As mentioned in Section 2.2 and Section 2.3, SnS_2_ nanoparticles were first prepared as the supporting host; then, a one-step *in situ* assembly method was used to synthesize NC@SnS_2_ samples by directly synthesizing/depositing CQDs on SnS_2_ surface, as shown in Scheme 1, instead of a two-step procedure: preparing CQDs and incorporating them onto SnS_2_ nanoparticles. This *in situ* assembly method simplified synthetic operation. In addition, the ratio of CA:UA was changed to modify the N-doping level in NC@SnS_2_ (corresponding to NC1@SnS_2_, NC2-4@SnS_2_, and NC3@SnS_2_). By fixing the ratio of CA:UA but changing the total amount of CA and UA, the CQD account in NC@SnS_2_ was modified as well (corresponding to the NC2@SnS_2_ series). For discussion convenience, CQDs (denoted as NC2 CQDs) were prepared as well, following a similar operation. It can be noticed from the following Section 2.4 that NC2 CQDs were N-doped ones, aiming at the desired performance.

### Synthesis of Reference Sample: NC2 CQDs

For performance comparison, reference CQDs were synthesized. A homogeneous solution was prepared by mixing CA and UA with deionized water (1.2 mmol:1.8 mmol in 25 ml). This solution was sealed in a Teflon autoclave and heated at 160^o^C for 4 h. After natural cooling, EtOH (50 ml) was added. The solid product was separated by centrifugation (×4,000 rpm) and washed by H_2_O/EtOH (1:1). The obtained solid powder was dried in a freeze dryer for 2 days and denoted as NC2 CQDs.

### Photocatalytic Performance Evaluation

The photocatalytic performance of NC@SnS_2_ samples was performed in a quartz reactor equipped with a cooling accessory via a water bath. The solar spectrum (excitation source) was simulated using a Xe lamp (PLS-SXE 300, 300 W) and a light filter (400 nm cutoff). The reaction temperature was set at 20^o^C (±2^o^C). NC@SnS_2_ (30 mg) was added into K_2_Cr_2_O_7_ aqueous solution (50 mg/L) and treated with an ultrasonic bath for 10 min. Then, this suspension was placed in dark for 2 h to allow full adsorption for photocatalyst. Finally, this mixture was exposed to the solar spectrum. For photoreaction monitoring, 0.3 ml of suspension was extracted each time (time interval = 300 s) and filtered off using a cellulose acetate membrane filter (pore size = 0.22 nm). The resulting solution was treated with diphenylcarbazide (as a chromogenic agent, λ_abs_ = 540 nm) and sent for UV-Vis absorption titration (Qu et al., 2013). Cr(VI) amount was proportional to the absorbance at 540 nm, so its photoreduction percentage was calculated by A/A_0_. Here, A_0_ was the initial absorbance value. It was noted that the adsorption of Cr(VI) on the surface of the photocatalyst is negligible (less than 2%), and modification of SnS_2_ with CODs has little effect on Cr(VI) adsorption.

The recycling performance of NC@SnS_2_ samples was evaluated as follows: after photoreaction, NC@SnS_2_ samples were filtered off and washed with plenty of freshwater. After being dried at 80^o^C for 12 h, the recycled NC@SnS_2_ samples were obtained.

### Electrochemical Performance Evaluation

Photo-generated charge transfer behavior was evaluated using an electrochemical workstation (CHI 760E). A three-electrode system was selected, with a Pt wire as the counter electrode, Ag/AgCl (saturated KCl) as the reference electrode, and NC@SnS_2_ as the working electrode (surface area = 0.17 cm^2^), using the electrolyte of Na_2_SO_4_, 0.2 M. The working electrode was prepared as follows: A mixture of NC@SnS_2_ (50 mg), deionized water (450 μL), EtOH (500 μL), and Nafion (5 μL) was treated with an ultrasonic bath for 10 min. Then, this mixture was spin-coated onto an FTO glass electrode (1.5 cm^2^ × 2.0 cm^2^) and heated at 400^o^C for 60 min under a pure N_2_ atmosphere. Mott–Schottky plots were sampled at 1,000 Hz, 2000 and 3,000 Hz. Electrochemical impedance spectra (EIS) were recorded at −1.4 V (vs. Ag/AgCl) within 0.1 Hz–10 kHz, using an AC sinusoidal perturbation (10 mV).

## Results and Discussion

### Morphology and Composition of NC@SnS_2_ Samples

**FIGURE 1:**
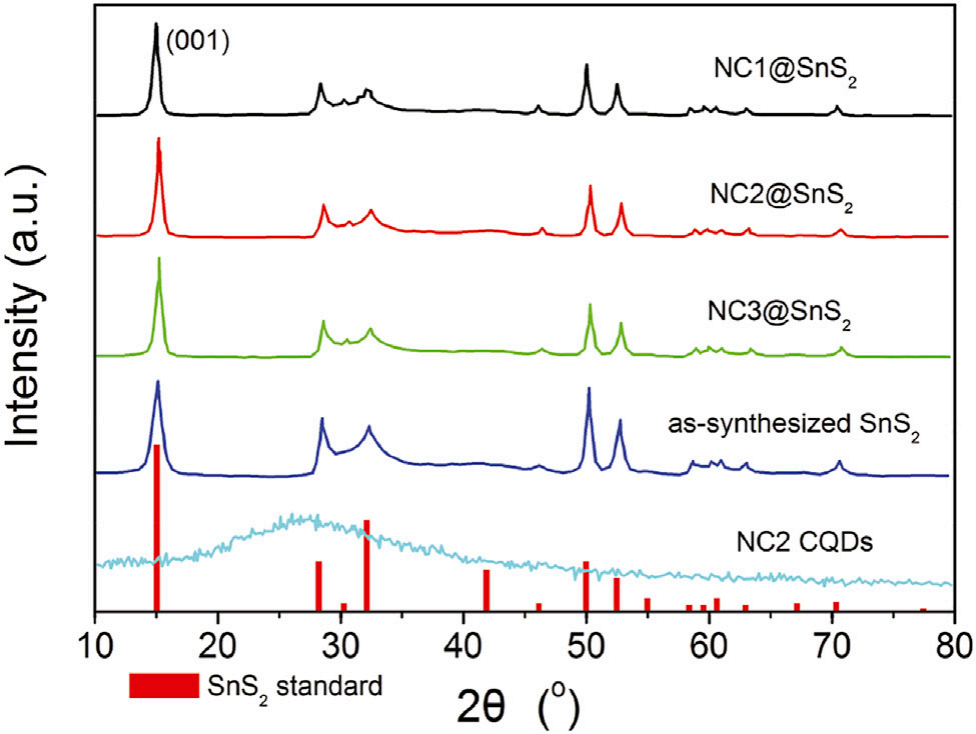
XRD patterns of SnS_2_ (standard JCPDS Card No. 23-0677), as-synthesized SnS_2_ in this work, and NC@SnS_2_.

As previously mentioned, NC@SnS_2_ was prepared by a one-step *in situ* assembly method, where the N atoms in CQDs are supposed to offer a midgap for the photogenerated charge carriers in SnS_2_. This midgap is supposed to improve electron mobility and thus promote the separation and transportation of photogenerated charge carriers. Simply speaking, here, the active site of NC@SnS_2_ should be the N-doped CQDs which capture excited electrons. The successful synthesis and assembly of NC2 CQDs, SnS_2_ nanoparticles, and NC@SnS_2_ samples are first discussed by comparing their XRD patterns. It is observed from Figure 1 that the primary diffraction peaks of NC@SnS_2_ samples are rather similar to those of as-synthesized SnS_2_ nanoparticles. Sharp diffraction peaks are observed, which confirms the high crystallinity of SnS_2_. All these diffraction peaks shall be indexed to a hexagonal SnS_2_ structure (JCPDS Card No. 23-0677). As for NC2 CQDs, a broad diffraction peak with 2θ = ∼27^o^ is observed, coming from the highly disordered C atoms (Xia et al., 2016). This broad diffraction peak, however, is not obvious in NC@SnS_2_ samples, owing to the low CQDs loading level in them. There is a notable difference in the relative intensity of (001) peak (vs. other diffraction peaks) between NC@SnS_2_ and as-synthesized SnS_2_ nanoparticles, which means an interaction between NC2 CQDs and SnS_2_ that causes variations in crystal size and preferred orientation. It appears that NC@SnS_2_ samples tend to grow along the c-axis direction, owing to below two reasons. First, there are enough functional groups on the CQD surface, which allows further nucleation. In addition, SnS_2_ nanoparticles tend to aggregate in the presence of NC2 CQDs, which will be confirmed by the following morphology analysis.

The synthesis of NC@SnS_2_ was finished by a controllable *in situ* assembly method. Here, NC2 CQDs were synthesized and directly loaded onto SnS_2_ nanoparticles under hydrothermal conditions through an electrostatic effect. To confirm the successful *in situ* assembly between SnS_2_ host and N-doped CQDs, their zeta potential values are determined as +4.30 mV for the SnS_2_ nanoparticles and −9.40 mV for NC2 CQDs (both dispersed in deionized water). There shall be strong electrostatic attraction between SnS_2_ host and N-doped CQDs, which leads to an assembly between them. The zeta potential value of a representative NC@SnS_2_ sample, NC2-4@SnS_2_, is determined as −4.50 mV, which is close to that of NC2 CQDs (−9.40 mV), compared to that of SnS_2_ nanoparticles (+4.30 mV). This is because SnS_2_ nanoparticles are covered by NC2 CQDs whose surface has negative groups such as -COO^-^. As a consequence, it is concluded that SnS_2_ nanoparticles have been successfully covered by NC2 CQDs.

**FIGURE 2:**
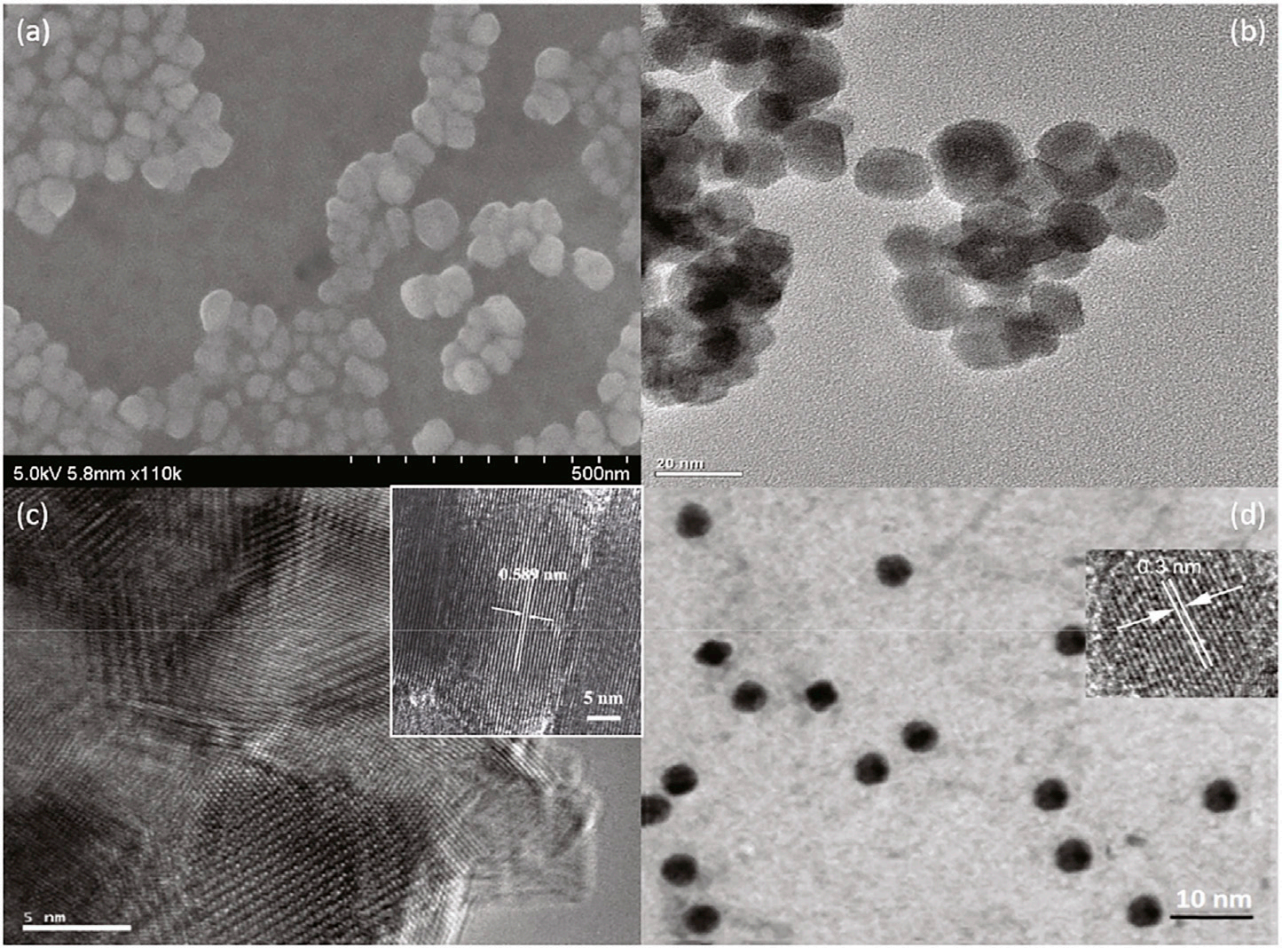
SEM **(A)**, TEM **(B)**, and HRTEM **(C)** of NC2-4@SnS_2_; TEM of NC2 CQDs **(D)**.

The successful *in situ* assembly is further analyzed via morphological analysis, which gives a visual understanding of NC@SnS_2_ samples. Here, the morphology of a representative NC@SnS_2_ sample, NC2-4@SnS_2_, is shown in Figures 2A–C, including its SEM, TEM, and HRTEM images. The TEM image of NC2 CQDs is shown for comparison (Figure 2D). As for NC2-4@SnS_2_, its SEM image suggests that the hexagonal morphology has been well preserved after loading NC2 CQDs. The average diameter of these NC2-4@SnS_2_ particles is 40 nm. As depicted by their TEM and HRTEM images, there are obvious lattice fringes in NC2-4@SnS_2_, with lattice spacing distances of 0.589 and 0.3 nm, respectively. The former one is attributed to the plane (001) from the SnS_2_ host. After comparing with the TEM details of NC2 CQDs shown in Figure 2D, the latter one is attributed to the plane (002) of graphic carbon from NC2 CQDs (Di et al., 2016; Li et al., 2016). The observation of these lattice fringes on NC2-4@SnS_2_ tentatively confirms the successful loading of CQDs on SnS_2_ nanoparticles. It is still observed that NC2 CQDs tend to stack on the SnS_2_ surface, showing a growth along the c-axis (surface vertical direction); this is because the abundant functional groups of NC2 CQDs endow themselves with high affinity for SnS_2_ nanoparticles, leading to the stacking along c-axis. This morphological analysis is consistent with the above XRD analysis. The *in-situ* assembly of NC2 CQDs on SnS_2_ nanoparticles is thus confirmed.

**FIGURE 3:**
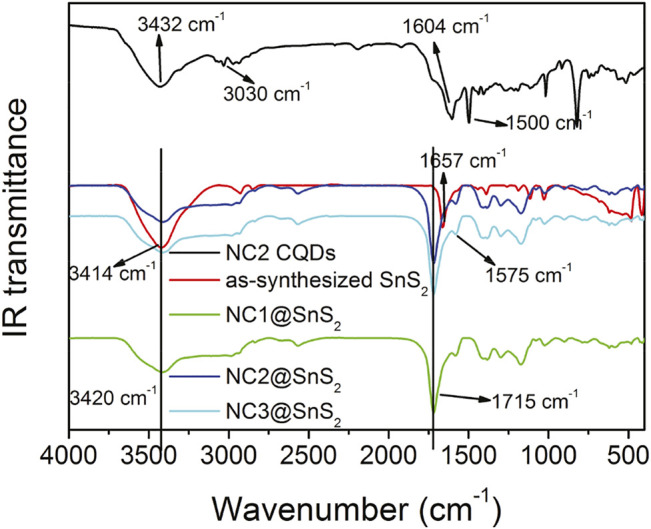
IR spectra of NC2 CQDs, SnS_2_ nanoparticles, and NC@SnS_2_ samples.

IR technique shall provide more details when analyzing the loading status of CQDs on SnS_2_ nanoparticles. IR spectra of NC2 CQDs, SnS_2_ nanoparticles, and NC@SnS_2_ samples are recorded and compared in Figure 3, so that the aforementioned hypothesis about CQDs stacking on SnS_2_ can be confirmed. The SnS_2_ IR spectrum is a simple one with only several peaks. The broad one around 3,414 cm^−1^ is attributed to the vibration of the –OH group (adsorbed water), while the sharp one at 1,657 cm^−1^ is attributed to the absorption of unsaturated bonds on the SnS_2_ surface (Tan et al., 2016). As for NC2 CQDs, there are several characteristic bands at 3,432 cm^−1^, 3,030 cm^−1^, 1,604 cm^−1^, and 1,500 cm^−1^, which are attributed to the vibrations from –OH (adsorbed water), N-H, C=N, and COO^−^ groups (Sun et al., 2013; Tan et al., 2016). After observing the IR spectra of NC@SnS_2_ samples, the IR bands from adsorbed water (3,420 cm^−1^) and C=N (1,575 cm^−1^) are still traced. More obviously, the IR band from unsaturated bonds on the SnS_2_ surface is blue-shifted to 1715 cm^−1^, compared to that of SnS_2_ nanoparticles (1,657 cm^−1^). This spectral shift suggests that the SnS_2_ surface has been covered by CQDs, which affects SnS_2_ unsaturated bonds and CQDs C=N bonds. Additionally, the IR absorption from CQDs COO^−^ groups is not observed in NC@SnS_2_ samples, suggesting that these COO^−^ groups have been grafted onto the SnS_2_ surface.

**FIGURE 4:**
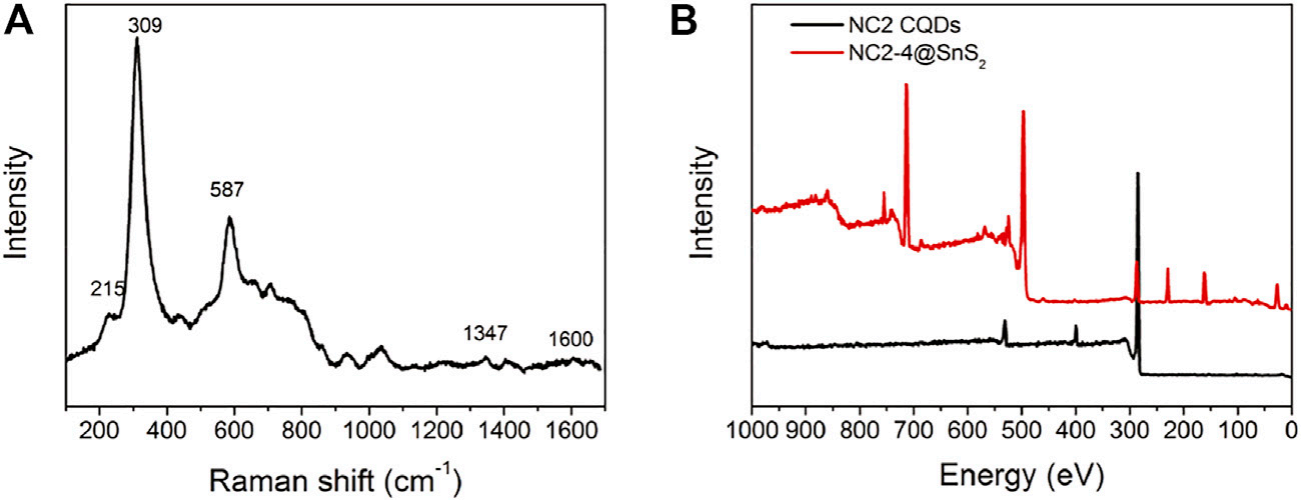
Raman **(A)** and XPS **(B)** spectra of NC2-4@SnS_2_.

Raman spectrum is a powerful tool to reveal sample surface chemistry. The Raman spectrum of NC2-4@SnS_2_ is shown in Figure 4A to get further information about NC@SnS_2_ samples. There are intense vibrational peaks of 215 cm^−1^, 309 cm^−1,^ and 587 cm^−1^, which are attributed to first-order E_g_, A_1g_, and second-order E_g_ of SnS_2_, while those of 1,347 cm^−1^ and 1,600 cm^−1^ are assigned as the Raman-active graphic (G) and defect (D) bands from CQDs (Muthulingam et al., 2015). In addition, the XPS survey spectra of NC2 CQDs and NC2-4@SnS_2_ are shown in Figure 4B. As for NC2 CQDs, characteristic peaks from carbon (1s), nitrogen (1s), and O (1s) atoms are clearly observed, which is consistent with the desired elemental composition of NC2 CQDs. After being loaded onto SnS_2_ nanoparticles, these peaks are all retrieved from the NC2-4@SnS_2_ XPS survey spectrum. In addition, characteristic peaks from Sn (3p1, 3p3, 3d, 4d) and S (2s, 2p) atoms are observed, confirming the elemental composition of NC2-4@SnS_2_.

Due to the higher electronegativity of nitrogen (3.0) than carbon (2.5), the doped N atoms shall serve as an electron acceptor. This hypothesis is approved by the following analysis on XPS binding energy. As shown by Supplementary Figures S1A–E (Supporting Information), the binding energy values of S 2p_1/2_ and S 2p_3/2_ (162.65 and 161.45 eV) in NC2-4@SnS_2_ are decreased by ∼0.4 eV, compared to those in SnS_2_ nanoparticles. Similarly, the N 1 s binding energy values of C-N (400.98 eV) and C=N (401.97 eV) in NC2-4@SnS_2_ are smaller than those in NC2 CQDs. The XPS peaks from C=O, C-OH, and C-O-O groups have been weakened or minimized in NC2-4@SnS_2_, compared to those in NC2 CQDs (Supplementary Figures S1B,C, Supporting Information), suggesting that these oxygen-containing groups are bonded with Sn. This statement is supported by the Sn binding energy analysis (Supplementary Figures S1E, Supporting Information). The binding energy values of Sn 3d_5/2_ and 3d_3/2_ in SnS_2_ nanoparticles are 487.1 and 495.5 eV. In NC2-4@SnS_2_, these values are decreased to 486.7 and 495.2 eV, indicating an interaction between NC2 CQDs and SnS_2_. The composite structure of NC@SnS_2_ is thus finally confirmed, where N-doped CQDs and hexagonal SnS_2_ crystals have been both identified. With the inserted midgap from CQDs, electron mobility is supposed to be improved, which promotes the separation and transportation of photogenerated charge carriers. Improved photocatalytic performance is thus expected from NC@SnS_2_.

### Photoreduction Performance of NC@SnS_2_ Samples

**FIGURE 5:**
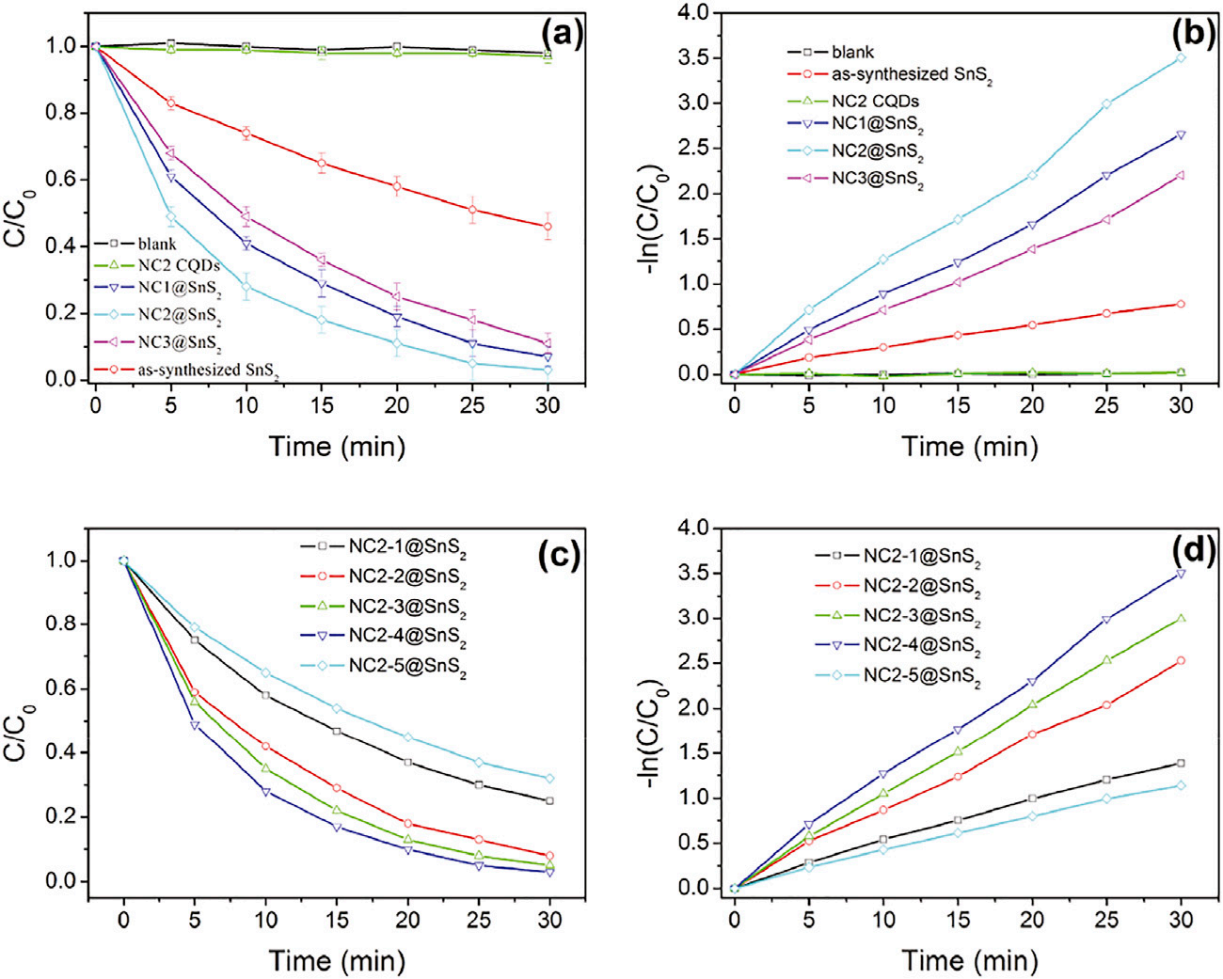
C/C_0_ and –ln (C/C_0_) values of Cr(VI) solution upon the presence of following photocatalysts: blank, NC2 CQDs, as-synthesized SnS_2_ nanoparticles, and NC@SnS_2_ samples.

Based on the above analysis and discussion, the successful synthesis of NC@SnS_2_ photocatalyst was confirmed. An improved photocatalytic performance was expected from NC@SnS_2_. Here, the photoreduction performance of NC@SnS_2_ samples was evaluated via Cr(VI) photoreaction. Photocatalytic performance comparison between NC@SnS_2_ samples, having different CQD amounts and different N doping levels was performed, with NC2 CQDs and as-synthesized SnS_2_ nanoparticles as reference groups. Here, diphenylcarbazide was used as a chromogenic agent, forming a purple-red compound in an acidic solution with absorption at 540 nm. Cr(VI) amount was proportional to the absorbance at 540 nm, so its photoreduction percentage (C/C_0_) was calculated by A/A_0_, where A_0_ was the initial absorbance value (Qu et al., 2013). It is observed from Figures 5A,B that the C/C_0_ value of pure Cr(VI) solution remains nearly constant (around 1.0) within the reaction time region 0-30 min, which means that Cr(VI) self-reduction is slim and neglectable. The existence of NC2 CQDs leads to a rather slim decrease (C/C_0_ = 0.97 at 30 min). Although NC2 CQDs have intense and broad absorption in the visible region (400–800 nm), the absorbed photons and their energy are wasted by thermal vibration, instead of participating in Cr(VI) photoreduction, which may be caused by the low efficiency of charge separation and transfer. An obvious Cr(VI) photoreduction is observed for as-synthesized SnS_2_ nanoparticles (C/C_0_ = 0.46 at 30 min). As for NC@SnS_2_ samples, their Cr(VI) photoreduction percentage is further increased, with C/C_0_ = 0.03 at 30 min for NC2-4@SnS_2_, for example. It is assumed that the conjugation planes in CQDs may accept the excited electrons from SnS_2_, which limits the recombination of photo-induced electron-hole pairs and thus improves the charge transfer separation efficiency of SnS_2_.

Based on the previoushypothesis, it is assumed that a suitable amount of CQDs with an optimal N doping level, which ensures an efficient electron capture, is required for optimal photocatalytic performance. This statement is supported by the photocatalytic performance comparison between NC@SnS_2_ samples with different CQD amounts and different N doping levels, as shown in Figures 5C,D. With more (NC1@SNS_2_) or less (NC1@SNS_2_) N-doping level in NC@SnS_2_, compared to NC2@SNS_2_, the photocatalytic performance is simultaneously weakened. This is because N atoms in CQDs help the electron capture procedure and accelerate charge carrier separation. On the other hand, the excess N atoms in CQDs may affect the CQDs conjugation plane and compromise charge carrier separation. As a consequence, there will be an optimal N-doping level, with strong enough electron capture and limited influence on the integrality of the CQD conjugation plane.

Even with the same N-doping level in NC2@SnS_2_ samples (NC2-1@SnS_2_, NC2-2@SnS_2_, NC2-3@SnS_2_, NC2-4@SnS_2_, and NC2-5@SnS_2_), their photocatalytic performance is different from each other, which means that the CQD amount plays an important role as well. Generally speaking, the photocatalytic performance of NC2@SnS_2_ is improved with increasing CQD amount. This observation is consistent with the aforementioned statement that CQDs help the electron capture procedure and accelerate charge carrier separation. Yet, NC2-5@SnS_2_ shows the worst photocatalytic performance among NC2@SnS_2_ samples, although its CQD amount is the highest. This observation is explained by the weakened light harvest since the excess CQDs may worsen SnS_2_ light absorption and thus compromise the generation of photo-induced electron-hole pairs. Apparently, the generation of photo-induced electron–hole pairs is controlled by SnS_2_ light harvest, not CQD light harvest. Excitation light shall first penetrate CQDs before reaching SnS_2_ and being absorbed by SnS_2_. In other words, SnS_2_ light harvest is limited by the outer CQDs, which denies a further photocatalytic efficiency improvement. This is a defect for the NC@SnS_2_ composite structure.

For a better understanding of the photoreduction dynamics of NC@SnS_2_ samples, C/C_0_ against reaction time is analyzed by a pseudo-first-order reaction, as described by Eq. 1, where *k* is the kinetic rate constant and t is reaction time:

$$\text{ln}\left(C/C_{0}\right)=kt.$$ (1)

The detailed fitting parameters are listed in Table 1. Good linear fitting is observed. The k-value of NC2-4@SnS_2_ is the highest one among NC@SnS_2_ samples.

**TABLE 1:** Fitting parameters of the following photocatalysts: blank, NC2 CQDs, as-synthesized SnS_2_ nanoparticles, and NC@SnS_2_ samples.

Photocatalyst	k (min^−1^)	R^2^
Blank	N/A	N/A
As-synthesized SnS_2_	0.0253	0.997
NC2 CQDs	N/A	N/A
NC1@SnS_2_	0.0869	0.998
NC21@SnS_2_	0.0460	0.998
NC22@SnS_2_	0.0818	0.999
NC23@SnS_2_	0.0991	0.999
NC24@SnS_2_	0.1144	0.998
NC25@SnS_2_	0.0379	0.999
NC3@SnS_2_	0.0711	0.998

### Photoreduction Mechanism of NC@SnS_2_ Samples

**FIGURE 6:**
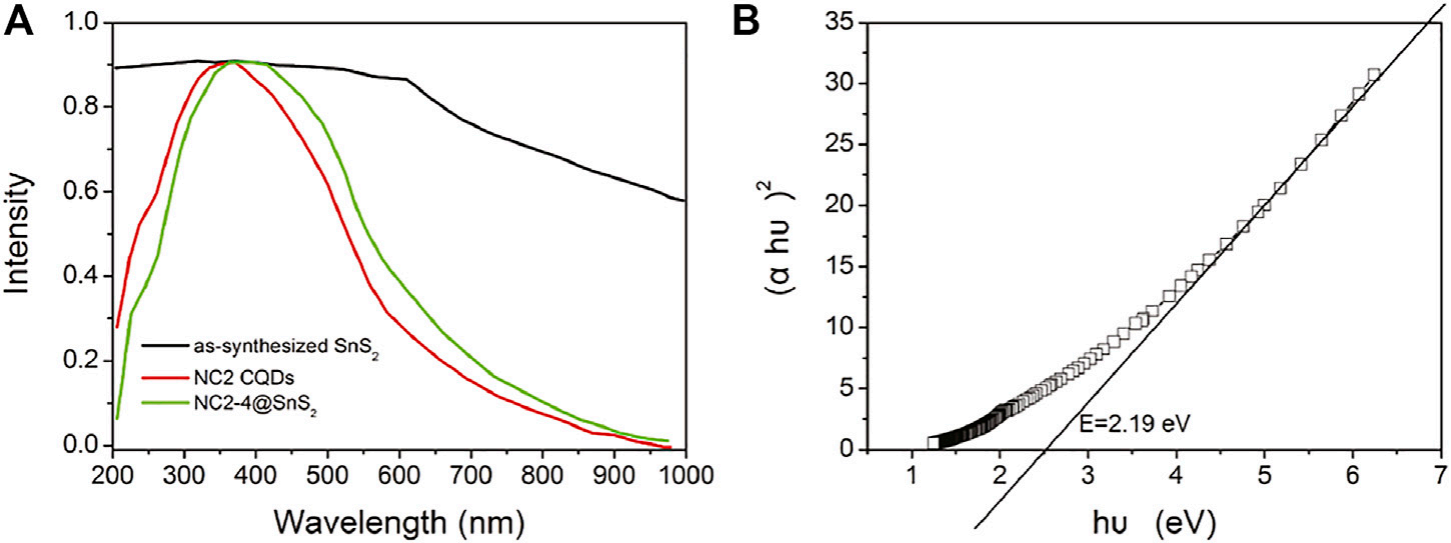
DSR spectra **(A)** of NC2 CQDs, as-synthesized SnS_2_ nanoparticles, and NC2-4@SnS_2_ and Tauc plots **(B)** of as-synthesized SnS_2_.

After demonstrating the photoreduction performance of NC@SnS_2_ samples, their mechanism is discussed via their UV-Vis diffuse reflectance spectra (DRS), as shown in Figure 6A. As for NC2 CQDs, a broad and intense absorption band covering UV and visible region from 200 to 800 nm is observed, with no vibronic progressions or secondary peaks, corresponding to p-p* transitions of aromatic C=C bonds, admixed with n-p* transitions of C=O bonds (Liang et al., 2013; Di et al., 2015). Regardless of the strong absorption of NC2 CQDs, their photocatalytic performance is neglectable, as mentioned previously, since there are numerous quenching centers in CQDs (-COO^-^, for example). As for the as-synthesized SnS_2_, a broad but weak absorption band covering the whole UV-Vis region (200-1,000 nm) is observed, which shall be attributed to the intrinsic band-to-band transitions in SnS_2_. After loading CQDs, NC@SnS_2_ DRS shows basically the DRS character of NC2 CQDs, with an obvious red shift of ∼25 nm. The close contact between NC2 CQDs and SnS_2_, as confirmed previously, shall neutralize the quenching centers in CQDs, which makes CQDs a photosensitizer with a midgap for SnS_2_, showing the aforementioned absorption redshift (Ran et al., 2015; Zhou et al., 2018). Upon photoexcitation of SnS_2_, the excited electrons can be trapped by this midgap level, which facilitates charge carrier transportation and depresses their recombination. As a consequence, an improved photocatalytic performance is observed for CQD-modified SnS_2_.

It has been reported that SnS_2_ has a direct bandgap structure; thus, its bandgap (E) is calculated by Tauc plots (Kubelk–Munk method), as described in Eq. 2 (Niu et al., 2014; Zhang et al., 2017):

$$\alpha \text{h}\nu =B{\left(h\nu -E\right)}^{1/2}.$$ (2)

Here, *α* is the absorption coefficient, h is the Planck constant, υ is frequency, and B is the fitting constant. With the DRS data on hand, the E value of SnS_2_ is calculated as 2.19 eV, as shown in Figure 6B. Since the conduction band (CB) level of SnS_2_ has been determined as −0.19 eV (against RHE), the valence band (VB) level can be calculated as 2.00 eV (Guo et al., 2018). When SnS_2_ is excited, its electron is transferred from VB to CB, resulting in a hole in VB and an excited electron in CB. The excited electron shall be captured by CQDs and then transferred to Cr(VI) to finish the photoreduction. As for the hole in VB, it shall be quenched by H_2_O, with molecular oxygen generated. This proposed mechanism is presented in Supplementary Figure S2 (Supporting Information).

**FIGURE 7:**
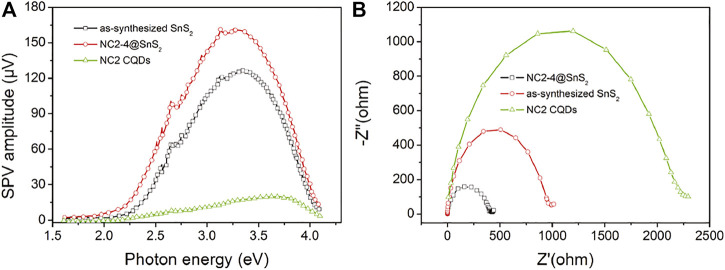
SPV **(A)** and EIS Nynquist plots **(B)** of NC2-4@SnS_2_, NC2 CQDs, and as-synthesized SnS_2_ nanoparticles.

To strengthen the previousstatement, surface photovoltage (SPV) and electrochemical impedance spectra (EIS) of NC2-4@SnS_2_, NC2 CQDs, and as-synthesized SnS_2_ nanoparticles are compared in Figure 7A. It is obvious that the onset energy of NC2-4@SnS_2_ (1.85 eV) is lower than those of NC2 CQDs (2.30 eV) and as-synthesized SnS_2_ nanoparticles (2.05 eV), indicating that NC2 CQDs decrease NC2-4@SnS_2_ excitation energy, which is consistent with the finding of their DRS comparison. Higher SPV values are observed for NC2-4@SnS_2_ (maximum = 161 μV) within photon energy region 2.25-3.75 eV, compared to those of NC2 CQDs (maximum = 21 μV) and as-synthesized SnS_2_ nanoparticles (maximum = 128 μV), which means that NC2 CQDs facilitate the charge carrier separation in NC2-4@SnS_2_. Meanwhile, the smallest radius is observed for the NC2-4@SnS_2_ EIS Nynquist plot, among the radii of NC2 CQDs and as-synthesized SnS_2_ nanoparticles, as shown in Figure 7B. It is thus confirmed that NC2 CQDs show the highest charge carrier separation efficiency, owing to the incorporation of CQDs, which favors Cr(VI) photoreduction. The positive effect of N-doped CQDs on improving the photocatalytic performance of NC@SnS_2_ is thus finally confirmed here.

### Recyclability and Durability Performance

**FIGURE 8:**
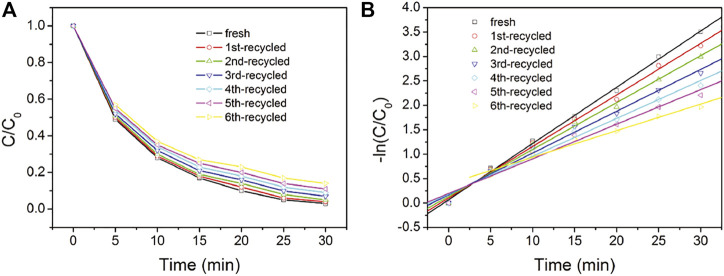
C/C_0_ **(A)** and –ln (C/C_0_) **(B)** values of Cr(VI) solution upon the presence of fresh and recycled NC2-4@SnS_2_.

**FIGURE 9:**
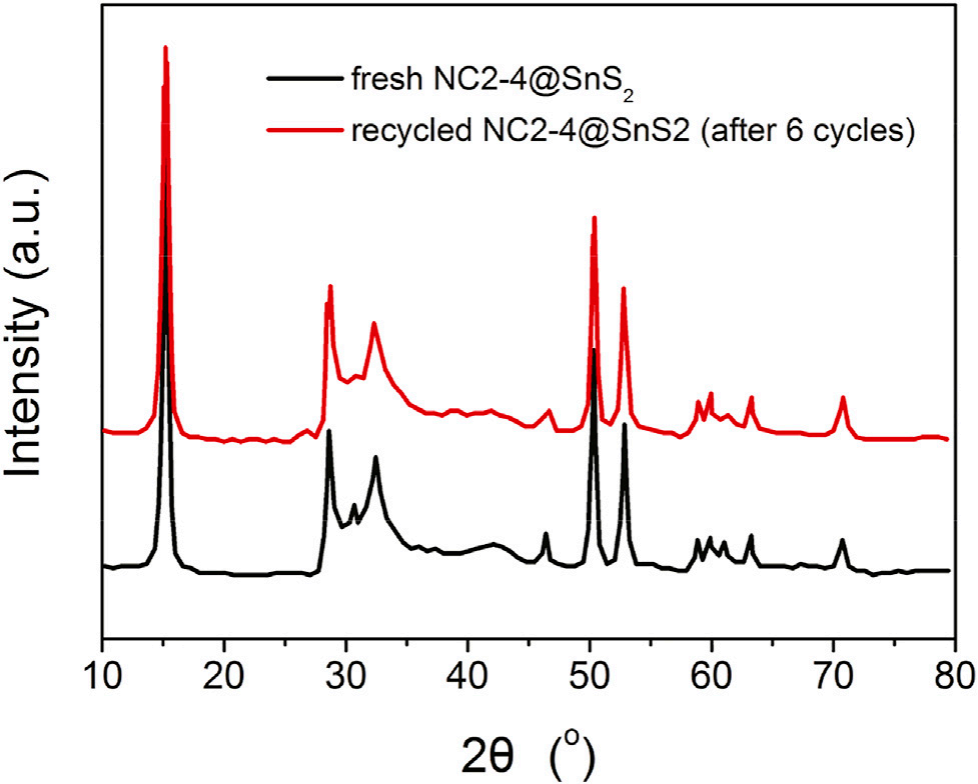
XRD patterns of fresh NC2-4@SnS_2_ and recycled NC2-4@SnS_2_ (after sixcycles).

The recyclability and durability performance of NC2-4@SnS_2_ is then discussed as follows: Figure 8 shows the photocatalytic performance of recycled NC2-4@SnS_2_. It is observed that the photocatalytic performance has been well preserved after the first five cycles, and then the photoreduction efficiency tends to decrease. Nevertheless, as shown in Figure 9, the XRD peaks of recycled NC2-4@SnS_2_ (after 6 cycles) are nearly identical to those of fresh NC2-4@SnS_2_, with no obvious shifts or intensity variations. As a consequence, the decreased photoreduction efficiency may be explained by the desorption of CQDs from NC2-4@SnS_2_. Considering that there is only Van der Waals' force, instead of strong chemical bonds, between CQDs and SnS_2_, it is highly possible that the CQDs are extracted and removed from the SnS_2_ surface, leading to decreased photoreduction efficiency. A down-bending tendency is observed for the recycled NC2-4@SnS_2_, especially for the sixth-recycled NC2-4@SnS_2_, which tentatively confirms the desorption of CQDs from NC2-4@SnS_2_.

## Conclusion

In conclusion, N-doped carbon quantum dots (CQDs) were prepared and loaded onto SnS_2_ nanoparticles via an *in situ* method. The resulting composite samples (NC@SnS_2_) were characterized by means of XRD, zeta potential, SEM/TEM, IR, Raman, and XPS to confirm their composite structure. Various N-doping levels and doping amounts were tried to optimize photocatalytic performance. The optimal NC@SnS_2_ showed improved photocatalytic performance, compared to pure SnS_2_, following a pseudo-first-order reaction with a kinetic rate constant of 0.1144 min^−1^. It was confirmed by diffuse reflectance spectra, surface photovoltage, and electrochemical impedance spectra that the loaded CQDs increased the charge carrier separation efficiency of SnS_2_. Good durability was observed for NC@SnS_2_, with at least five full cycles. On the other hand, the charge carrier separation efficiency of NC2-4@SnS_2_ is still yet to be satisfied. For further efforts, the holes in SnS_2_ VB shall be neutralized quickly to improve charge carrier separation efficiency. In addition, the loaded CQDs served only as a midgap for SnS_2_ but showed no contribution to SnS_2_ absorption. The intrinsic absorption of SnS_2_ should be enhanced to improve its absorption of excitation light. Finally, the durability of NC@SnS_2_ should be improved as well. Our primitive suggestion is to replace N-doped CQDs (carbon quantum dots) with N/P-codoped CQDs (Pan et al., 2022). It has been reported that P atoms have a conjugation interaction with SnS_2_ (forming $\text{Sn}<\begin{array}{c} \text{S}\\ \text{S} \end{array}>\text{P}$ structure) (Li, 2001; Yang et al., 2018). In this case, the bonding between CQDs and SnS_2_ shall be increased, hoping to get a better durability.

## Data Availability

The original contributions presented in the study are included in the article/Supplementary Material; further inquiries can be directed to the corresponding authors.
