# Corrigendum: Modifying SnS_2_ with carbon quantum dots to improve photocatalytic performance for Cr(VI) reduction

**DOI:** 10.3389/fchem.2022.1097501

**Published:** 2022-11-25

**Authors:** Weidong Li, Jianping Qiu, Haihong Jin, Yuanyuan Wang, Dandan Ma, Xinxiang Zhang, Huayun Yang, Fangyuan Wang

**Affiliations:** ^1^ Zhejiang Normal University Xingzhi College, Jinhua, China; ^2^ Hangzhou Normal University Qianjiang College, Hangzhou, China; ^3^ Zhejiang Hongyi Environmental Protection Technology Co., Ltd., Hangzhou, China; ^4^ Environmental Engineering Corporation of Zhejiang Province, Hangzhou, China; ^5^ Zhejiang Tianchuan Environmental Science and Technology Co., Ltd., Hangzhou, China; ^6^ Zhejiang Normal University, Jinhua, China

**Keywords:** photocatalyst, SnS_2_, Cr(VI), carbon quantum dots, photoreduction

In the original article, there was an error in the affiliation numbers of authors Weidong Li and Jianping Qiu. The correct affiliation numbers appear above.

The authors apologize for this error and state that this does not change the scientific conclusions of the article in any way. The original article has been updated.

## Publisher’s note

All claims expressed in this article are solely those of the authors and do not necessarily represent those of their affiliated organizations, or those of the publisher, the editors and the reviewers. Any product that may be evaluated in this article, or claim that may be made by its manufacturer, is not guaranteed or endorsed by the publisher.

