# Supplementary material for: Modifying SnS2 With Carbon Quantum Dots to Improve Photocatalytic Performance for Cr(VI) Reduction
Source: Front Chem. 2022 Jun 22;10:911291. doi: 10.3389/fchem.2022.911291 (PMC9257045; doi:10.3389/fchem.2022.911291)

Supplementary Material

Figure S1a. High resolution XPS (C 1s) of NC2 CQDs and NC2-4@SnS_2_.


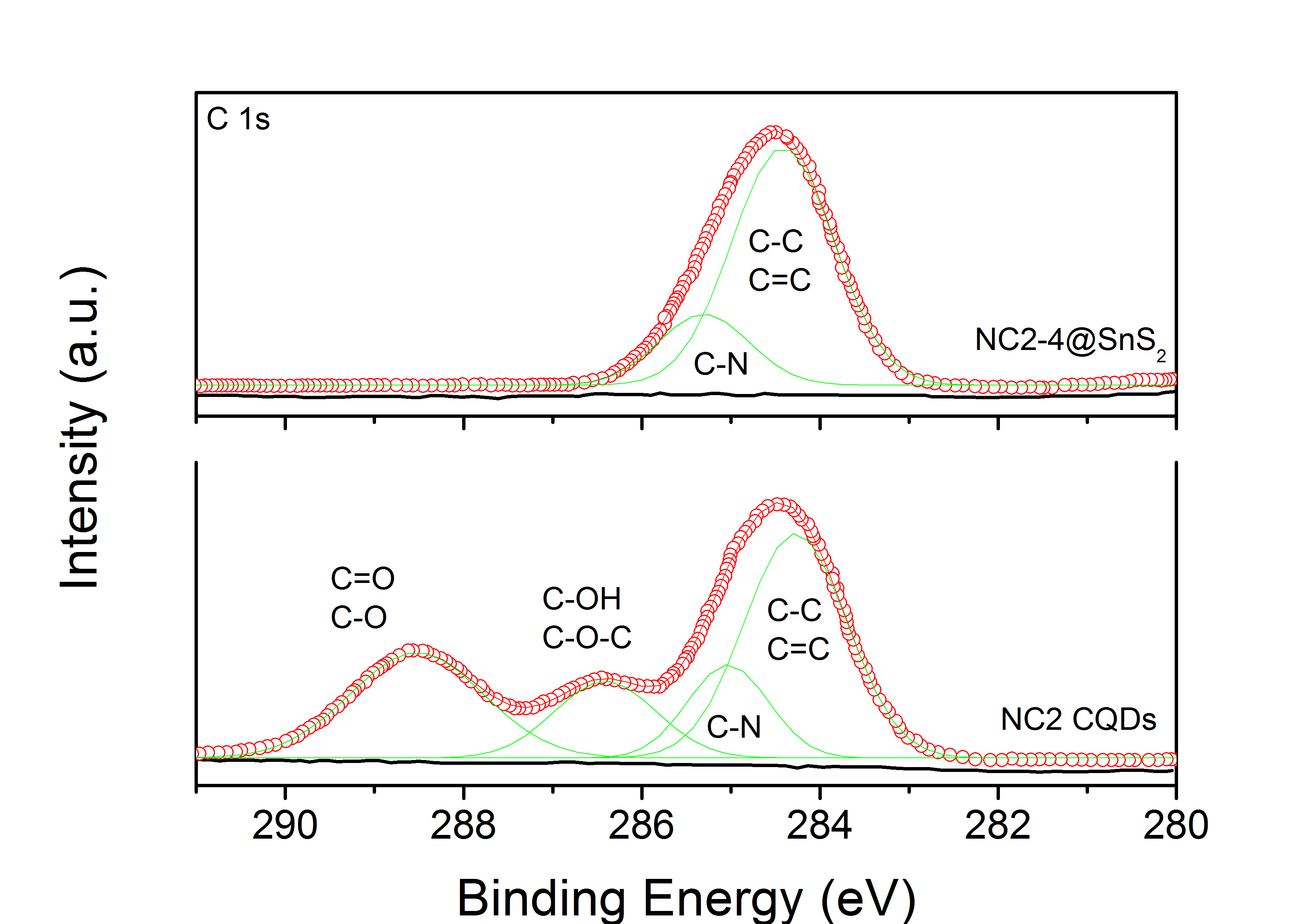


Figure S1b. High resolution XPS (N 1s) of NC2 CQDs and NC2-4@SnS_2_.


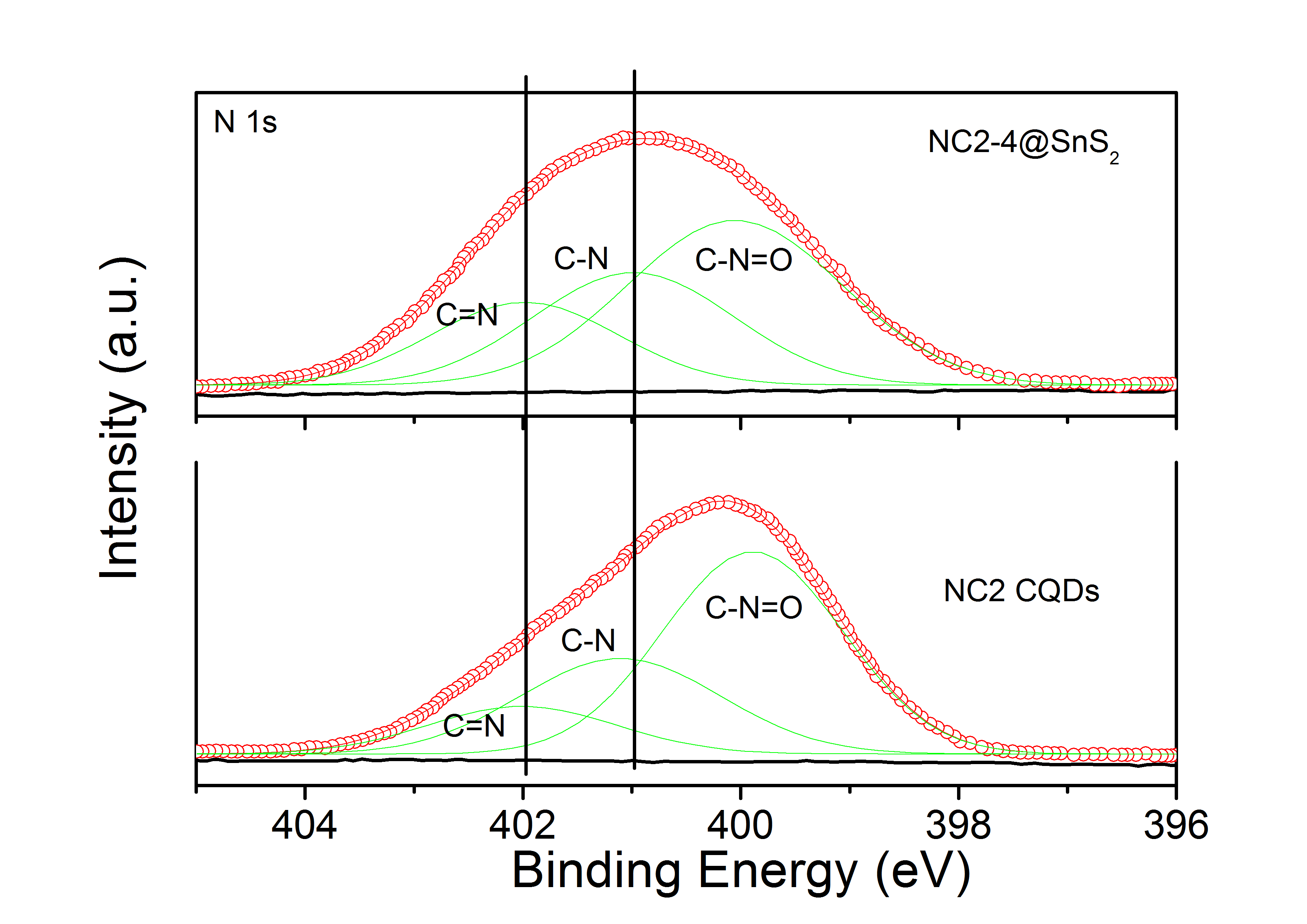


Figure S1c. High resolution XPS (O 1s) of NC2 CQDs and NC2-4@SnS_2_.


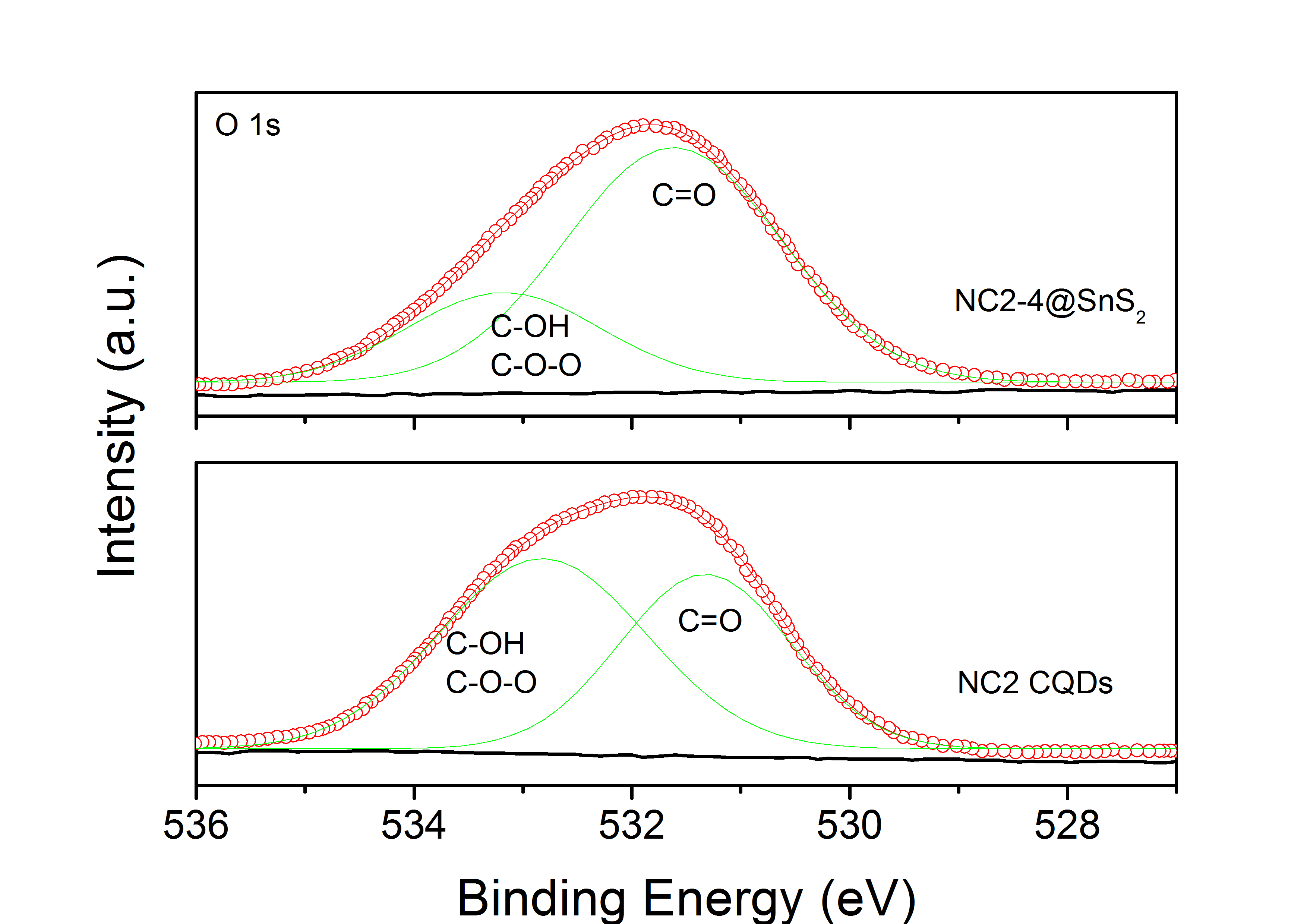


Figure S1d. High resolution XPS (S 2p) of SnS_2_ and NC2-4@SnS_2_.


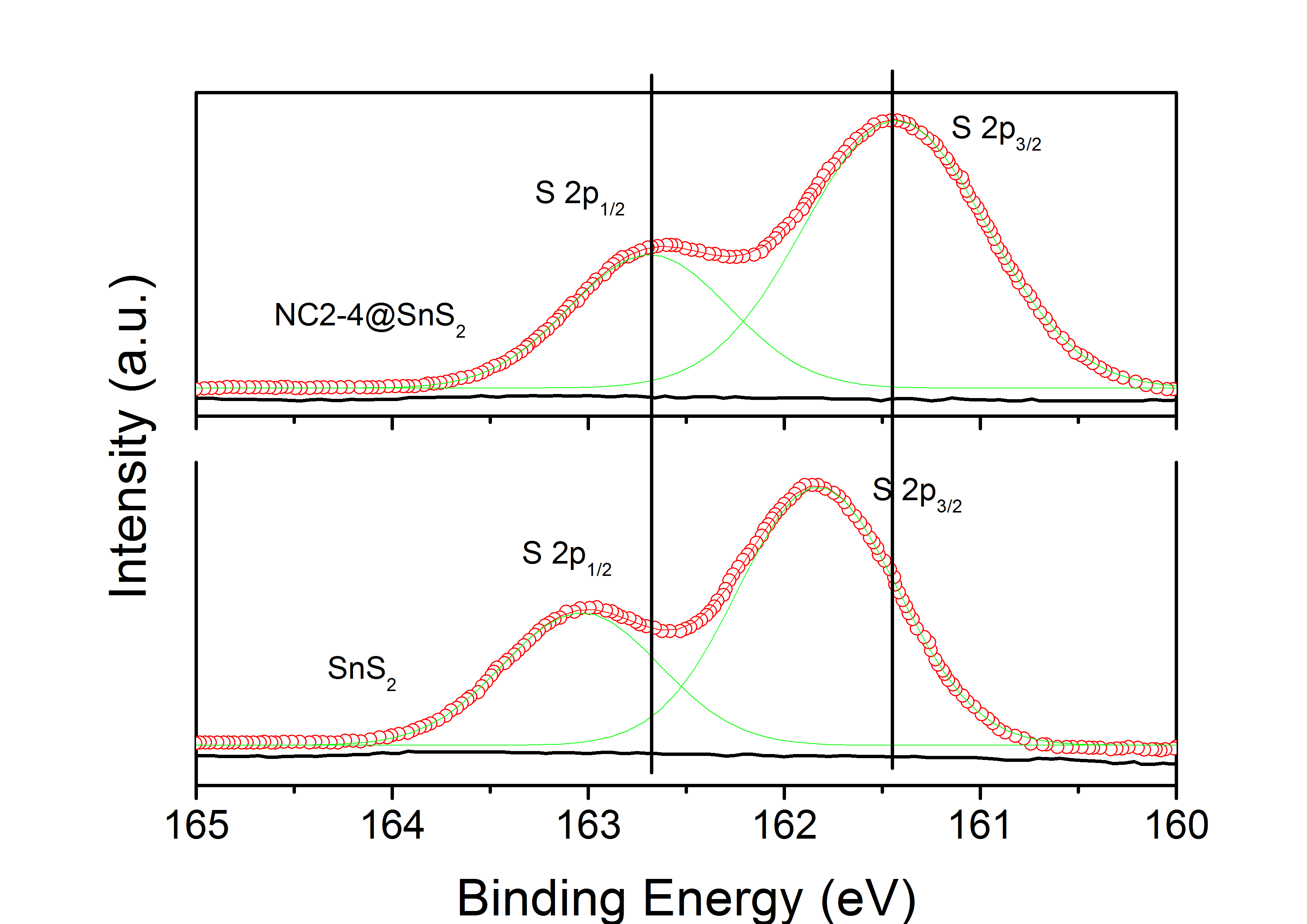


Figure S1e. High resolution XPS (Sn 3d) of SnS_2_ and NC2-4@SnS_2_.


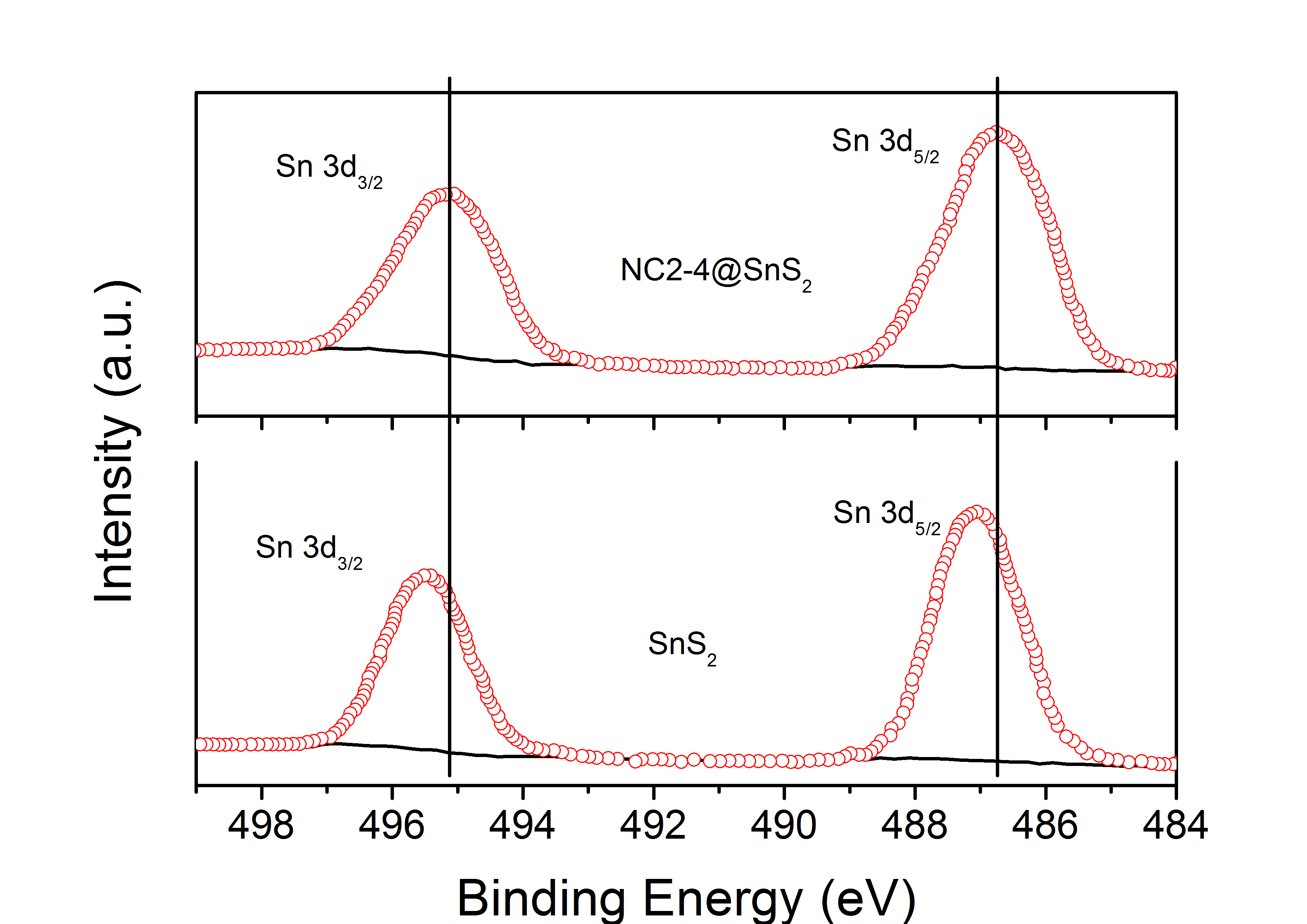


Figure S2. A possible photocatalytic mechanism of NC@SnS_2_.


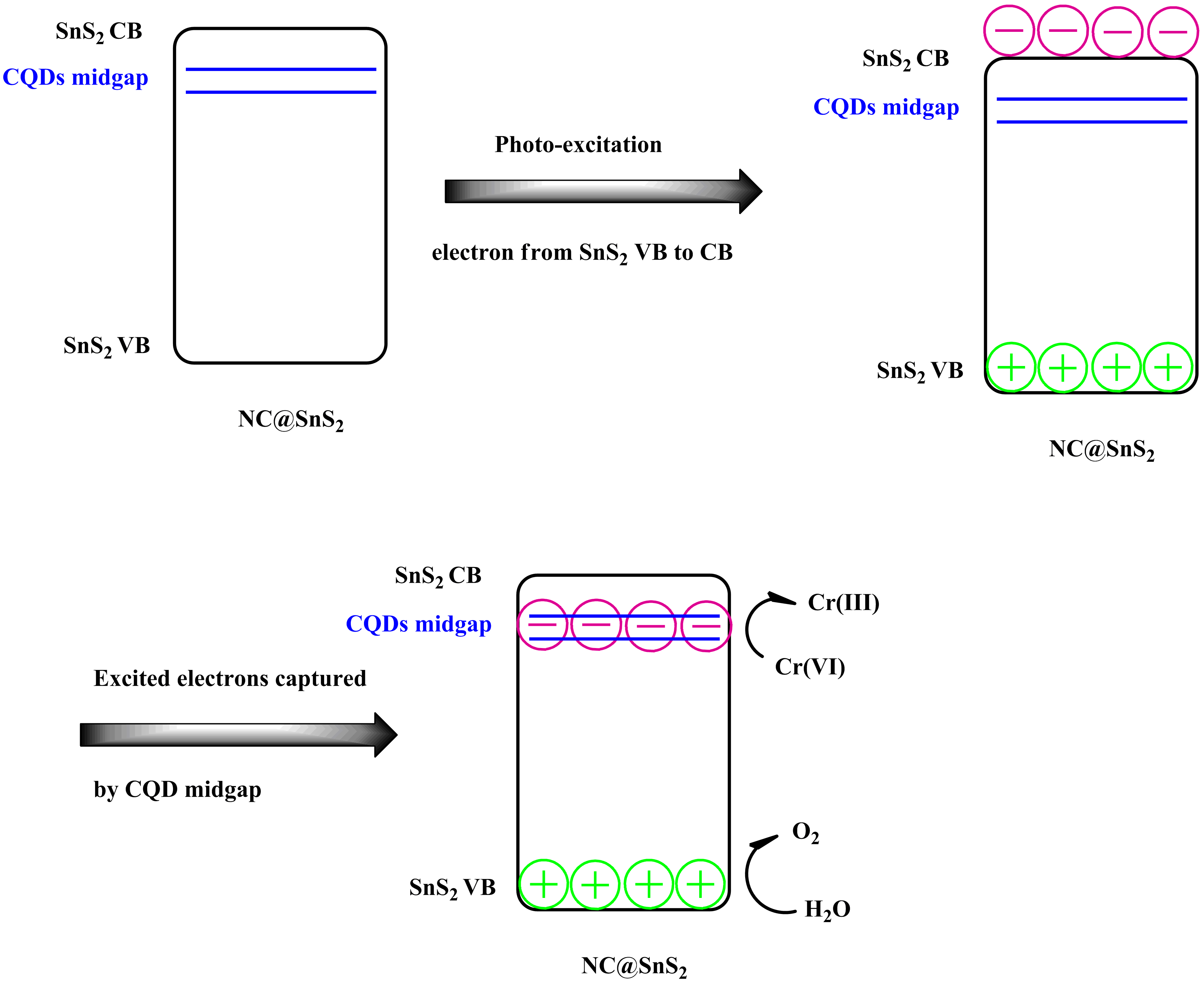

Supplement: Supplementary file 1 [file Table1.DOCX]
